# Effectiveness of a Saffron and Withania Supplement on Mood in Women With Mild-to-Moderate Anxiety During the COVID-19 Lockdown

**DOI:** 10.1155/2024/3661412

**Published:** 2024-11-11

**Authors:** Cristina Pages-García, M. Cristina De Almagro, Jorge Ruiz-Moreno, Roser De Castellar

**Affiliations:** ^1^Obstetrics and Gynecology Department, Hospital Universitario, Toledo, Hospitales de Madrid HM IMI Toledo, Unidad de Suelo Pélvico, Toledo, Spain; ^2^Parc Científic de Barcelona, Laboratorios Ordesa S.L., Barcelona 08028, Spain; ^3^Head of Biometry, Mixestat S.L., Barcelona 08021, Spain; ^4^Clinical Research Unit, Medical Affairs Department, Laboratorios Ordesa S.L, Barcelona 08038, Spain

**Keywords:** anxiety, *Crocus sativus*, depression, quarantine, *Withania somnifera*

## Abstract

**Background:** A nutritional supplement based on medicinal plants (saffron and ashwagandha), tryptophan, and vitamin B6 could contribute to alleviating/improving mood and associated disorders. The aim of this study was to evaluate the potential benefits of this combination supplement. During the study period, participants underwent a period of forced home confinement due to the COVID-19 pandemic, which represented an unexpected impact factor.

**Methods:** This open-label prospective trial enrolled a cohort of female employees who reported mild to moderate anxiety. The primary objective was to evaluate changes in the level of anxiety using the adapted Hamilton Anxiety Rating Scale (HARS) after 12 weeks of regular supplementation with Safromotive (two tablets daily, for 12 weeks). The secondary objectives were to evaluate health-related quality of life (HRQoL) and tolerability.

**Results:** In total, 46 women with a mean age of 45.0 (6.5) years were included. A statistically significant improvement in HARS was observed, with a 7.5-unit decrease from baseline to 12 weeks (*p*  < 0.0001) and from 4 to 12 weeks of supplement intake (*p*=0.0058). However, no significant changes were found during the lockdown period (between weeks 8 and 12 of the study). No relationship was found between women's sociodemographic characteristics and the HARS total score. A significant reduction in the HRQoL questionnaire score of 1.2 units was observed between baselines and 12 weeks of treatment (*p*=0.0273). At the end of the study, 78.6% of the women reported consistency the supplement intake during the study course.

**Conclusion:** This nutritional supplement composed of saffron, ashwagandha, tryptophan, and vitamin B6 appears to improve anxiety and HRQoL, but confinement could have impacted the evolution of the outcome.

## 1. Introduction

Mental disorders are among the leading causes of health-related morbidity [1]. In 2019, the Global Burden of Diseases, Injuries, and Risk Factors Study conducted in 204 countries revealed that the two most disabling mental disorders were depressive and anxiety disorders, both of which are ranked among the top 25 leading causes. In addition, these disorders are more prevalent in women, and along with headaches, are the three leading causes of disability-adjusted life years [2]. In 2020, a global pandemic, derived from the emergence of a new coronavirus called COVID-19, contributed to high mortality rates due to the lack of knowledge about the new virus and the lack of resources during the first months of the pandemic, which led to significant social alarm [3]. All of society was subjected to stressful and traumatic circumstances that could have an impact on mental health [4–6], which led to increases of 27.6% and 25.6% in cases of depression and anxiety, respectively [1]. Short- and long-term psychiatric symptoms have been observed not only in infected or front-line health care workers but also in the general population [7, 8]. Nonessential workers were oriented to perform work activities from home; they were more stressed due to a heavier workload and intensity, combining both professional life and family care (minors, elderly individuals, and pets) [9]. In addition, social life and physical activity were restricted, with a negative influence on mental health [8, 9]. Uncertainty associated with the pandemic also generated anxiety and distress [10] with an unequal impact, affecting women more severely than men [1].

It is common for people with psychological symptoms to self-medicate, even though psychotropic drugs must be prescribed. The use of tranquilizers carries numerous risks; they generate tolerance and may be associated with dependence and, ultimately, addiction. Furthermore, they can cause undesirable side effects (memory and concentration problems) and associated adverse events (accidents at work, falls, etc.) [11, 12]. Numerous medicinal plants have traditionally been used for anxiety disorders and depression because they produce fewer adverse events [13]. Saffron (*Crocus sativus* L.) has multiple recognized therapeutic properties, including antidepressant, anxiolytic, and stress-reliever effects [14]. Withania (*Withania somnifera* L. Dunal), also known as “Ashwagandha” or “Indian Ginseng,” is characterized by adaptogenic activity and the ability to reduce physical and psychological symptoms associated with stress and anxiety [15, 16]. Likewise, tryptophan, an essential amino acid that is abundant in meat, fish, eggs, milk, whole grains, and certain fruits, seeds, and nuts, is related to mood, behavior, and cognitive function. Tryptophan is a precursor of serotonin and vitamin B3 and is involved in the release of neurotransmitters such as dopamine, noradrenaline, and beta-endorphin [17]. The proper tryptophan catabolic pathway requires the assistance of vitamin B6 [18], which also helps to alleviate the symptoms caused by depression and anxiety [19].

Many systematic reviews and meta-analyses have focused on the impact of the COVID-19 pandemic on mental health, mainly among front-line health professionals. However, studies of psychosocial aspects and work capacity among nonessential workers are lacking [9].

Therefore, this open label, prospective intervention trial was conducted with the purpose of evaluating the potential benefits of a nutritional supplementation based on medicinal plants (saffron and ashwagandha), tryptophan, and vitamin B6 to alleviate or improve mood and associated disorders, such as anxiety. The main objective of this study was to evaluate the effect of nutritional supplementation for 12 weeks on anxiety symptoms. The secondary objectives were to evaluate health-related quality of life (HRQoL), adherence to treatment, and tolerability.

Coincidentally, during the active period of this study, the participants were nonessential workers who underwent a period of forced home confinement due to the COVID-19 pandemic, which represented an unexpected impact factor that could have influenced the evolution of the results presented here.

## 2. Materials and Methods

### 2.1. Study Design

An open label, prospective intervention trial was conducted among a cohort of female employees of the manufacturer laboratory who reported mild-to-moderate anxiety as part of the Internal Employee Benefits Program to improve their quality of life. No remuneration was offered to the participants, and no extra costs were incurred by their participation. The participants were offered a nutritional supplement (Safromotive), which was based on medicinal plants (saffron, 30 mg/2 tablets and ashwagandha, 150 mg/2 tablets), tryptophan (600 mg/2 tablets), and vitamin B6 (1.4 mg/2 tablets) to alleviate or improve mood and associated disorders, such as anxiety. These ingredients and their doses were selected on the basis of published clinical evidence [20–31]. The ingredients included L-tryptophan, hydroxypropyl methylcellulose, Ashwagandha extract (*W. somnifera* root, standardized to 5% withanolides), dicalcium phosphate, microcrystalline cellulose, saffron extract (*C. sativus* flower, standardized to 2% safranal and 12% safromotivins), magnesium stearate, carrot concentrate (*Daucus carota* root), and pyridoxine chlordidrate (vitamin B6). The dosage was two tablets daily, taken preferably at breakfast, for 12 weeks. Recruitment lasted 2 weeks, and the study period was 12 weeks (from December 2020 to March 2021) and had four assessment timepoints (initial, 4-, 8-, and 12-week controls). The included participants were women aged 25–55 years with mild-to-moderate anxiety according to the Hamilton Anxiety Rating Scale (HARS) (scoring between 8 and 24 points), without active or chronic diseases that could interfere with their overall health status (kidney, heart, or liver disease; metabolic or endocrine disorders; active oncological processes; etc.) or who had any oncological disease or treatment in the past year. All the participants provided written informed consent to participate. Women were excluded if they were pregnant or intended to become pregnant, had eating disorders, or had experienced a stressful life circumstance in the last 6 months. Women receiving anxiolytic or antidepressant treatment were excluded only if they had changes in their usual treatment regimen in the previous 2 months. At the baseline assessment timepoint, those diagnosed with a severe level of anxiety were recommended to consult their health care providers and were excluded from the study. All the investigators who designed the study and conducted the assessments and surveys were health care professionals. All the study assessments were conducted via videoconference.

On January 7, 2021, an unplanned stressful event that simultaneously affected all participants occurred. The regional government decreed home confinement for 4 weeks for all municipalities. All participants continued their work activities from home during this period.

### 2.2. Study Endpoints and Variables

Demographic and clinical characteristics were recorded at enrollment. The primary objective was to evaluate changes in the level of anxiety using the adapted HARS after 12 weeks of regular supplementation. The Spanish validated version of the adapted HARS [32, 33] was completed by health care professionals via video calls. This scale consists of 14 items scored from 0 (not at all) to 4 (makes normal life impossible), with total scores indicating mild (≤17), mild-to-moderate (18–24), and moderate-to-severe (25–30) anxiety. The HARS includes two subscales for somatic and psychic items.

The secondary objectives included assessing the impact of the supplement on participants' HRQoL at the four assessment timepoints, estimating participants' adherence to treatment, and collecting information on the tolerability of the treatment. To measure HRQoL, the four COOP–WONCA charts were used in a Spanish-validated version [34], covering the domains of Physical Fitness, Feelings, Daily Activities, and General Health (each chart comprises a domain that scores: 1 = very good, 2 = good, 3 = moderate, 4 = poor and 5 = very poor). Changes were considered moderate when the score shifted by ≥1 and <2 points and relevant when it was ≥2 points. The Pain and Changes in Health Status domains were excluded because participants were not starting from a disease situation. Additionally, the Social Activities domain was excluded due to a “ceiling effect” in people with mild impairment of quality of life [35]. These were also completed by health care professionals via video call. Each COOP/WONCA chart has independently demonstrated appropriate reliability and validity for determining HRQoL [36, 37]. Finally, the Haynes–Sackett test, a validated self-reported adherence questionnaire, was administered to participants to assess compliance with the nutritional supplementation regimen [34]. Additionally, factors that could positively or negatively affect the expected effects of the supplement were identified.

### 2.3. Statistical Analysis

Continuous variables are expressed as the means and standard deviations (SDs) or minimum and maximum values, whereas categorical variables are expressed as absolute and relative frequencies. For comparisons between timepoints, the means or medians of changes with their 95% confidence intervals (CIs) were calculated. The main analysis compared changes that occurred between baseline and completion of the 12-week study period. The percentage change, defined as ([[current assessment value − initial assessment value]/initial assessment value] × 100), was calculated along with the CI. An improvement was considered when a negative change in the means, medians, or percentages occurred between the current assessment and baseline. Additionally, 95% CIs were calculated for event proportions. The normality of variable distributions was tested using the Shapiro‒Wilk test. The results were accompanied by hypothesis tests using the *t*-test or the Wilcoxon signed-rank test for paired data and contrast for proportions. The relationships between baseline variables and total scores on the Hamilton scale at 12 weeks were graphically evaluated. The analysis of the “COOP–WONCA” domains was performed by dimension, whereas the sum score analysis of the four charts was only exploratory. Statistical significance was set at *p*  < 0.05. STATA 17 was used for data management and statistical analysis, and PASS 2021, V21.0.2, was used to calculate the power of the study (0.99). The results of the power of the study to detect an effect size (Cohen's D for paired data, defined as the mean of the difference divided by the SD of the difference) were based on a 2000-sample Monte Carlo simulation from the normal distribution. Changes between baseline and the 12-week assessment timepoint and the upper limit of the 95% CI of SD estimated in the study sample were considered.

### 2.4. Sample Size Calculation

Considering a sample size of 28 women, a difference between 12 weeks of treatment and a baseline total anxiety score of −7.5, and a SD of 8.12 (corresponding to the upper limit of the 95% CI), the study provides a power of 0.99 to detect an effect size of 7.5/8.12 = 0.92, with a true type I error of 0.055 (95% CI: 0.045, 0.064).

### 2.5. Ethical Aspects

The participants provided written informed consent to record possible changes in their mood and quality of life. The protocol was favorably evaluated by the Bioethics Committee of the University of Barcelona (November 2019, Institutional Review Board: IRB0003099), and the project had the scientific and clinical advice of a physician specializing in gynecology. All the investigators were health professionals (pharmacists and doctors) exclusively working at the manufacturer's laboratory, who maintained confidentiality. The Human Resources Department and the rest of the company were blinded to those who had attended the appointment and who had ultimately participated.

## 3. Results

In total, 54 women applied for the study, and 46 were included. The mean age (SD) was 45 years (6.5 years), and the majority were salaried employees. The sociodemographic and clinical characteristics of the participants were collected and are detailed in Table 1.

Overall, 28 participants (60.9%) completed the study period, and 18 individuals (39.1%) discontinued participation, with an average duration of 48 days (range: 12–68 days), for the following reasons: loss to follow-up due to loss of employment (*n* = 11), loss of the supplement (*n* = 1), poor tolerance due to supplement aftertaste (*n* = 3), and intercurrent illness (*n* = 3).

### 3.1. Primary Endpoint: Changes in Anxiety Symptoms

Effectiveness in anxiety management was reflected in a significant decrease in the HARS total score by 7.5 units from baseline to 12 weeks of treatment (95% CI: −9.9, −5.2; *p*  < 0.0001). The score reduction between these two timepoints was also significant for both the psychic subscale (−3.9 units, 95% CI: −5.4, −2.4, *p*  < 0.0001) and the somatic subscale (−3.6 units, 95% CI: −4.9 and −2.4; *p*  < 0.0001).

**Figure 1:**
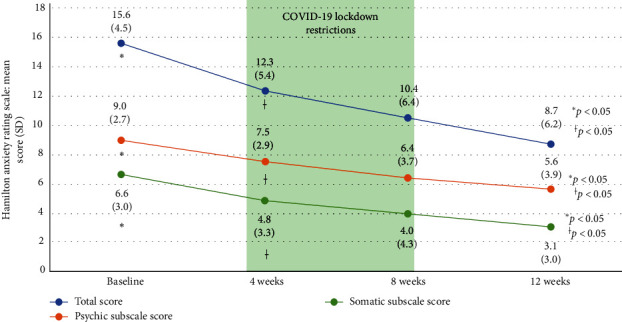
Evolution of the Hamilton test score over the study course. The scores are provided as the mean values (standard deviations). Significant differences were found from baseline to week 12 (*⁣*^*∗*^*p*  < 0.05), from baseline to week 4, and from week 4 to week 12 (^†^*p*  < 0.05) during the study period. SD, standard deviation.

A significant score decrease between the 4-week assessment timepoint and baseline was found for all three HARS measures (total: −3.6 units [95% CI: −5.2, −2.0; *p*=0.0001]; psychic: −1.7 [95% CI: −2.7, −0.8, *p*=0.0006]; and somatic: −1.9 [95% CI: −3.0, −0.8, *p*=0.001]). Additionally, three HARS measures comparing the 12-week and 4-week assessment timepoints were significantly lower (total: −3.4 units [95% CI: −5.8, −1.1; *p*=0.0058]; psychic: −2.1 [95% CI: −3.5, −0.7, *p*=0.0048]; and somatic: −1.4 [95% CI: −2.6, −0.2, *p*=0.0277]). In contrast, there were nonsignificant reductions in the HARS total score (−1.8 units [95% CI: −4.0, 0.4; *p*=0.0969] and somatic subscale score (−0.5 units [95% CI: −1.6, 0.6; *p*=0.3875]) between the 8-week and 4-week periods of treatment, but a significant reduction in the psychic subscale score (−1.32 units [95% CI: −2.58, −0.06; *p*=0.0408]) was detected. Finally, between the 12- and 8-week assessment timepoints, the total HARS (−1.4 units [95% CI: −3.1, 0.2; *p*=0.0811]) and both subscales, psychic (−0.7 units [95% CI: −1.8, −0.4; *p*=0.1963]) and somatic (−0.7 units [95% CI: −1.6, −0.1; *p*=0.0716]), did not show significant score reductions (Figure 1).

The mean HARS scores for the total, psychic, and somatic subscales were significantly lower at 12 weeks than at baseline or at the 4-week assessment timepoint (Table 2).

The proportions of women who improved, regardless of the degree of reduction in their global, psychic, and somatic HARS scores at the 12-week period of treatment compared with baseline, were 86% (95% CI: 67%–96%; *p*  < 0.0001), 82% (95% CI: 63%–94%; *p*  < 0.0001), and 86% (95% CI: 67%–96%; *p*  < 0.0001), respectively.

### 3.2. Secondary Endpoints

#### 3.2.1. Factors That Affect the Expected Effects of the Complement

No relationships were found between baseline variables such as age, education, family composition, or lifestyle and the HARS total score.

#### 3.2.2. Quality of Life

**Figure 2:**
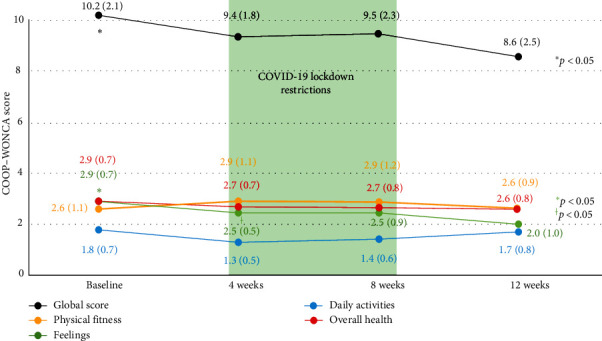
COOP/WONCA: Evolution of the global score and chart score. The scores are provided as the mean values (standard deviations). Significant differences were found from baseline to week 12 (*⁣*^*∗*^*p*  < 0.05) and from week 4 to week 12 (^†^*p*  < 0.05) of the study period.

The Feelings chart of the HRQoL scale revealed a significant reduction of 1 unit from baseline to 12 weeks of the study period (95% CI: −1.42, −0.56; *p*  < 0.0001) as well as a significant reduction of 0.48 units between the 4- and 12-week assessment timepoints (95% CI: −0.83, −0.13; *p*=0.0095). No statistically significant differences were observed in the other subscales (Physical Fitness, Daily activities, and Overall Health), although a trend toward a reduction in scores over time was observed (Figure 2).

#### 3.2.3. Adherence

Among all the participants, 94.6% reported not forgetting to take the supplement at 4 weeks, 83.3% at 8 weeks, and 78.6% at 12 weeks.

#### 3.2.4. Tolerability

The percentages of women who reported any discomfort potentially related to supplement intake at the 4-, 8-, and 12-week assessment timepoints were 21.6%, 6.7%, and 10.7%, respectively. The unexpected events reported during the study are detailed in Table 3.

#### 3.2.5. Subjective Perception

A total of 46% of the participants perceived an improvement in their anxiety, 86% reported that the supplement was “easy to take,” and 68% reported that they wanted to extend their supplement intake.

## 4. Discussion

Depression and anxiety are among the leading causes of health loss worldwide, and a significant increase due to the COVID-19 pandemic has been reported [1]. Uncertainty about the evolution of the pandemic as well as the strict measures of social isolation summed to the disruption of work life were associated with increased stress, anxiety, depression, and sleep problems. The relationship between quarantine and anxiety has been identified in many publications, and the longer the confinement is, the greater the level of anxiety, with a strong correlation between both variables (*r* = 0.152, *p*  < 0.001). Correlations have also been observed between quarantine and depression (*r* = 0.115, *p*  < 0.001) and between quarantine and stress (*r* = 0.125, *p*  < 0.001) [38]. During the course of our study, municipal confinement was decreed for several weeks (between the 4th and 8th weeks, ±1 week), and the COVID-19 pandemic led to company restructuring. These were unforeseen stressful events that affected all participants simultaneously. Nonetheless, it was decided not to stop the study course, although there was a significant number of dropouts after 4 weeks of treatment. Most of these dropouts were participants who reported a “loss of interest” due to this circumstance.

Currently, in our environment, there is increasing use of herbal supplements to alleviate psychological problems, especially among middle-aged women [39], who are the most at-risk population for anxiety disorders. For example, saffron has been reported in previous studies to be useful in the management of stress and anxiety during prolonged isolation. In fact, some authors estimate that its efficacy in the management of depression is comparable to that of some drugs [40]. A meta-analysis of randomized clinical trials demonstrated that saffron intake significantly reduces anxiety and depression scores on the Beck Depression Inventory (weighted mean difference [WMD]: −4.9; 95% CI: −6.6, −3.14) and Beck Anxiety Inventory (WMD: −5.3; 95% CI: −8.3, −2.3) but not on the Hamilton Depression Rating Scale (HDRS) or HARS [41]. Additionally, a random-effects meta-analysis performed by Marx et al. [42] revealed a large positive effect size in favor of saffron in reducing anxiety compared with placebo control conditions (*g* = 0.95, 95% CI: 0.27–1.63, *p*  < 0.006), which was used at the same doses as in our study (30 mg/day of saffron). In fact, with the correction for publication bias, the effect size of saffron supplementation was even larger (*g* = 1.40, 95% CI: 0.60–2.150). Several mechanisms have been suggested to be involved in the antidepressant properties of saffron. It has been proposed that saffron could have various effects, such as anti-inflammatory, antioxidant, neuroprotective, and hypothalamus–pituitary–adrenal-modulating effects [43]. A substantial amount of research has demonstrated that inflammation and immune dysfunction play significant roles in the development of depression and anxiety disorders [44, 45]. As reviewed by Shafiee et al. [43], the anti-inflammatory properties of saffron have been demonstrated in animal models for a wide range of inflammatory diseases, such as arthritis [46], asthma [47], and colitis [48]. In terms of antioxidant effects, high levels of oxidative stress and reduced antioxidant potential are closely related to anxiety and depression [49, 50], and a meta-analysis revealed that increased levels of oxidative stress markers are associated with depression [51]. It has been extensively reported that saffron possesses antioxidant properties. For example, in rats with hyperlipidemia, saffron and crocin were found to have significantly positive effects on several oxidative markers [52]. In terms of neuroprotection, it has been suggested that low levels of brain-derived neurotrophic factor (BDNF) could play a key role in the pathogenesis of depression and anxiety disorders [53]. Several studies with animal models have confirmed the neuroprotective properties of saffron and its components, which exhibit antidepressant-like effects by increasing several proteins (such as BDNF) related to antidepressant activity in the hippocampus [54, 55]. Finally, it has been proposed that saffron could have an impact on the hypothalamic–pituitary–adrenocortical (HPA) system. Hyperactivity of the HPA system as well as elevated levels of cortisol have commonly been observed in depression [56] and are usually reversed by antidepressant therapy [57]. Studies have shown that stress cannot alter corticosterone levels in rats or mice treated with saffron water extract, safranal, or crocin [58, 59]. Moreover, crocin injection significantly reduces plasma corticosterone levels in rats subjected to chronic restraint stress [60]. Interestingly, in another study by Fukui,Toyoshima, and Komaki [61] exposure to saffron odor for 20 min led to decreased cortisol levels in both the follicular and luteal phases in 35 female participants, accompanied by a reduction in anxiety symptoms.

Regarding ashwagandha, it is not very clear how it exerts its action; partly, it may be due to its antioxidant activity, but it can also be due to its GABAergic activity [62]. *γ*-Aminobutyric acid (GABA) is an inhibitory neurotransmitter whose function is to decrease neuronal function and prevent overexcitation, which leads to insomnia and nervousness [63]. The GABAergic activity of *W. somnifera* on GABA-A and GABA-*ρ*1 ionotropic receptors could explain its efficacy in the treatment of insomnia [62]. In fact, studies suggest that it contains an ingredient with GABA-mimetic action [64]. It has been proposed that changes in GABAergic signaling play crucial roles in the underlying causes of major depressive disorders [65]. A randomized, double-blind, controlled clinical trial that studied its efficacy on different parameters revealed a significant decrease in HARS in the experimental group after 10 weeks of treatment (baseline: 23.6 [22.6–24.6] vs., 10 weeks: 18.5 [17.4–19.6]) [15]. Similarly, a meta-analysis of 12 studies with 1003 participants aged 25–48 years revealed a beneficial effect of ashwagandha supplementation on both stress (standardized mean difference (SMD): −1.75; 95% CI: −2.29, −1.22; *p*=0.005; I2 = 83.1%) and anxiety (SMD: −1.55, 95% CI: −2.37, −0.74; *p*=0.005; I2 = 93.8%) [16]. Moreover, in a double-blind randomized placebo-controlled trial, an ashwagandha extract administered at a dosage of 125 mg/day significantly decreased the modified anxiety Hamilton scale (mHAM-A), the plasma biomarkers cortisol and CRP, blood pressure, and heart rate [66]. This trial supports the effectiveness of Safromotive in our study, which is a product that contains 150 mg/day ashwagandha extract.

Despite this, supplementation with saffron, ashwagandha, tryptophan, and vitamin B6 was effective against mild–moderate anxiety in women aged 25–55 years. The response to the supplement was significant and objectively measurable by the changes in the level of anxiety observed throughout the study period and after 4 weeks. Although a high percentage of participants reported improvement in HARS (85% between the beginning and the end of the study period) at each assessment timepoint, this improvement in anxiety levels was increasingly milder over time, and the changes at 8 weeks were no longer statistically relevant; this can be explained by the fact that these were participants with mild–moderate baseline states, but it also suggests the impact of a stressful environmental factor such as the period of confinement, which would have had a negative impact on the quality of life of these confined workers, who saw their activities and social relationships reduced, and which would explain the lack of changes in these dimensions of the COOP_WONCA scale. These results were consistent with those obtained for the COOP/WONCA scale, where a significant improvement was also detected, specifically in the feelings domain. Considering that these were women without health problems, the COOP–WONCA scale detected a moderate or greater improvement (>1 point) in perceived quality of life but not in the domains of physical fitness, daily activities, and general health. For the latter, the scale loses its ability to assess response to change (<1 point). Moreover, these domains could be directly affected by confinement. Additionally, no significant differences were reported in the HARS scores between the 8- and 12-week study periods, possibly because due to the same reason. During this timeframe, the capacity for improvement triggered by the supplement is expected to decrease, leading to a certain plateau effect on the QoL scores, as can also be observed through the analysis of the COOP_WONCA scale. Studies have shown that a reduction in physical exercise and restrictions in daily activities resulting from confinement affects the perception of QoL. The COOP_WONCA scale applied to subjects without underlying pathologies has a risk of a ceiling effect [35]. However, for its Feelings dimension, a significant improvement was detected.

Sociodemographic factors are associated with anxiety disorders. Women and some marital statuses (widowers, unmarried, or divorced people) have a higher prevalence of anxiety disorders [67]. In our study, only four women lived alone, and the other 42 women lived with others. The group of women who lived alone was not large enough to apply any statistical comparison with the other group.

This study focused on healthy women with mild-to-moderate levels of anxiety, 37% of whom lived with their children. Some epidemiological studies on the COVID-19 lockdown have revealed higher anxiety levels in women with children than in women without children, which may be particularly related to difficulties in balancing personal life, working, and caring for children [68].

As a part of the study procedures, women were encouraged to inform their general practitioner about their participation. Good physician‒patient communication was considered essential for conciliating nutritional supplement intake into a patient's treatment plan.

This study has some intrinsic limitations due to its design. First, a control arm was absent, and there was a relevant number of losses due to the COVID-19 crisis. Another limitation is that a variable of other coping mechanisms for anxiety was included, so we cannot isolate the effect of the supplement on anxiety. Among its strengths, this study captured the beneficial impact of supplement intake under exceptionally stressful circumstances due to the COVID-19 pandemic.

## 5. Conclusions

In conclusion, the nutritional supplement composed of saffron, ashwagandha, tryptophan, and vitamin B6 appears to be well tolerated and to have a positive effect on women with mild-to-moderate anxiety, as well as on their HRQoL; however, confinement could have an impact on the evolution of the outcome. Further studies with larger numbers of individuals of various genders and ages are needed to confirm these results.

## Figures and Tables

**Table 1:** Baseline sociodemographic and clinical characteristics.

Characteristic	*N* = 46
Sociodemographic data	
Age (years), mean (SD)	44.96 (6.49)
Education, *n* (%)	
Higher	33 (71.7)
Secondary	13 (28.3)
Family composition, *n* (%)	
Couple with children	24 (52.2)
Couple without children	10 (21.7)
Alone with children	7 (15.2)
Alone	4 (8.7)
Other composition	1 (2.2)
Employment status, *n* (%)	
Salaried	45 (97.8)
Self-employed	1 (2.2)
Anthropometrics	
Weight (kg), mean (SD)	64.0 (10.9)
Height (cm), mean (SD)	165.9 (5.9)
Healthy lifestyles, *n* (%)	
Regular physical activity^a^	30 (65.2)
Sufficient vegetable consumption^b^	30 (65.2)
Nonsmokers^c^	40 (86.9)
Low alcohol consumption^d^	46 (100.0)
Pathological history, *n* (%)	
Obesity	1 (2.2)
Arthropathies	2 (4.4)
Depression	2 (4.4)
Respiratory disease	2 (4.4)
Other	3 (8.3)

Abbreviation: SD, standard deviation.

^a^Regular physical activity: At least 30 min active walking five times/week or sports two times/week.

^b^Sufficient daily consumption of fruit and vegetables (at least two servings of fruit and three servings of fresh and/or cooked vegetables).

^c^Not a regular smoker or a former smoker for more than 2 months.

^d^No or very sporadic consumption of alcoholic beverages.

**Table 2:** Percentage of change of Hamilton Anxiety Rate Scores between assessment timepoints.

Time intervals	*N*	Change (%)	CI 95%	*p* value
Hamilton Anxiety Rating Scale (global)				
12 weeks—baseline	28	−46.61^a^	−60.93, −32.30	<0.0001
12 weeks–4 weeks	27	−26.09^a^	−44.42, −7.75	0.0071
12 weeks–8 weeks	27	−13.84	−33.16, 5.48	0.1529
8 weeks–4 weeks	28	−13.11	−32.40, 6.17	0.1744
4 weeks—baseline	37	−22.39^a^	−33.60, −11.19	0.0003
Hamilton anxiety rating—psychic subscale				
12 weeks—baseline	28	−45.30^a^	−65.75, −24.85	<0.0001
12–4 weeks	28	−25.69^a^	−42.67, −8.72	0.0045
12–8 weeks	27	−10.67	−29.92, 8.56	0.2644
8–4 weeks	28	−12.18	−34.30, 9.94	0.2686
4 weeks—baseline	37	−16.26^a^	−27.40, −5.13	0.0054
Hamilton anxiety rating—somatic subscale				
12 weeks—baseline	28	−67.53^a^	−91.08, −43.99	<0.0001
12–4 weeks	25	−50.00^a^	−82.23, −17.77	0.0437
12–8 weeks	20^b^	−19.48	−45.21, 6.25	0.1296
8–4 weeks	26	−7.60	−37.62, 22.42	0.6067
4 weeks—baseline	37	−24.85^a^	−41.72, −7.98	0.0050

Abbreviation: CI, confidence interval.

^a^Nonparametric test: Wilcoxon test, *p*  < 0.05.

^b^There were some noncomputable subjects with a change value = 0.

**Table 3:** Reported unexpected events.

Unexpected events	Related to supplement intake	Number of cases (*n*)		
		4 weeks	8 weeks	12 weeks
Diuresis increase (related to higher water intake)	Yes (higher water intake)	2	—	—
Unpleasant herbal aftertaste	Yes	5	1	1
Slight feeling of nausea	Yes	2	—	—
Insomnia	Possibly	1	1	—
Constipation	Possibly	—	—	1
Fluid retention	Probably	—	—	1
Total	—	8	2	3

## Data Availability

The data that support the findings of this study are available from the corresponding author (Roser De Castellar) upon reasonable request.
